# Cryo-EM structure of the human sodium-chloride cotransporter NCC

**DOI:** 10.1126/sciadv.add7176

**Published:** 2022-11-09

**Authors:** Jing Nan, Yafei Yuan, Xuemei Yang, Ziyang Shan, Huihui Liu, Feiwen Wei, Wei Zhang, Yanqing Zhang

**Affiliations:** ^1^ Shanghai Fifth People’s Hospital, Fudan University, and Shanghai Key Laboratory of Medical Epigenetics, International Co-laboratory of Medical Epigenetics and Metabolism (Ministry of Science and Technology), Institutes of Biomedical Sciences, Fudan University, Shanghai 200032, China.; ^2^ Beijing Frontier Research Center for Biological Structure, Beijing Advanced Innovation Center for Structural Biology, Tsinghua-Peking Joint Center for Life Sciences, School of Life Sciences, Tsinghua University, Beijing 100084, China.; ^3^ Warshel Institute for Computational Biology, School of Life and Health Sciences, The Chinese University of Hong Kong, Shenzhen 518172, Guangdong, China.

## Abstract

The sodium-chloride cotransporter NCC mediates the coupled import of sodium and chloride across the plasma membrane, playing vital roles in kidney extracellular fluid volume and blood pressure control. Here, we present the full-length structure of human NCC, with 2.9 Å for the transmembrane domain and 3.8 Å for the carboxyl-terminal domain. NCC adopts an inward-open conformation and a domain-swap dimeric assembly. Conserved ion binding sites among the cation-chloride cotransporters and the Na2 site are observed in our structure. A unique His residue in the substrate pocket in NCC potentially interacts with Na1 and Cl1 and might also mediate the coordination of Na2 through a Ser residue. Putative observed water molecules are indicated to participate in the coordination of ions and TM coupling. Together with transport activity assays, our structure provides the first glimpse of NCC and defines ion binding sites, promoting drug development for hypertension targeting on NCC.

## Teaser

Cryo-EM structure of the human sodium-chloride cotransporter NCC promotes drug development for hypertension targeting on NCC.

## INTRODUCTION

Encoded by genes of the solute carrier 12 (SLC12) family, the electroneutral cation-chloride cotransporters (CCCs) mediate coupled movement of Na^+^ and/or K^+^ with Cl^−^ across the membrane, playing essential roles in a variety of physiological processes, such as cell volume regulation, ion homeostasis, and neuronal excitability (*1*–*3*). CCCs can be divided into two functional branches (*4*). The first branch is Na^+^-dependent cotransporters, composed of two Na^+^-K^+^-Cl^−^ cotransporters, NKCC1 and NKCC2, and one Na^+^-Cl^−^ cotransporter, NCC, responsible for ion import. The second branch is Na^+^-independent cotransporters, which encompass four K^+^-Cl^−^ cotransporters, KCC1 to KCC4, exporting ions out of cells. CCCs are widely expressed in the brain, kidney, heart, etc. Dysregulation of CCCs causes multiple human diseases, such as salt-wasting disorders and neurological diseases (*5*–*7*).

NCC, encoded by the *SLC12A3* gene, is expressed in the distal convoluted tubule in the kidney, responsible for sodium reabsorption and playing a pivotal role in extracellular fluid volume and blood pressure control (*8*–*11*). Malfunction of NCC leads to renal Na^+^ waste and hypotension, causing Gitelman syndrome, a widely reported inherited kidney disease, depicted by metabolic alkalosis and hypokalemia, along with urinary calcium, hypocalcemia, and hypomagnesaemia (*5*, *12*). Increased NCC activity leads to familial hyperkalemic hypertension (*13*–*15*). Hypertension, a leading risk factor for myocardial infarction, stroke, and congestive heart failure, affects billions of individuals worldwide (*16*). Thiazide-sensitive NCC has been treated as the drug target for hypertension by the first-line clinic drugs, thiazide diuretics (*17*).

Structures of NKCC1 and KCC1-4 share similar overall architectures, most of which were solved in an inward-open state (*18*–*26*). In addition, two structures in the outward-facing state were recently solved: NKCC1 bound with bumetanide (*27*) and KCC1 bound with VU0463271 (*28*). For SLC12 family, apart from the monomeric structure of mouse KCC4 in nanodiscs (*24*), all the resolved structures present as a dimer. In these structures, the conserved binding sites of K^+^ and two Cl^−^ have been observed, as well as the Na2 binding site found in NKCC1, although with relatively weak density (*18*, *27*).

While KCCs and NKCC1 structures revealed the architectures and ion binding sites of K^+^ (or Na^+^-K^+^)–transporting CCCs, the structure and ion-cotransport mechanism of NCC, which is Na^+^ dependent and uniquely does not transport K^+^, remain unknown. Here, we present the cryo–electron microscopy (cryo-EM) structure of human NCC. Combined with transport assays and structural comparisons, our results ultimately define the Na^+^ and Cl^−^ binding sites of NCC; provide clues for ion selectivity, coupling, and translocation; and promote further drug development for hypertension and Gitelman syndrome targeting on NCC.

## RESULTS

### Structure determination

**Fig. 1.**
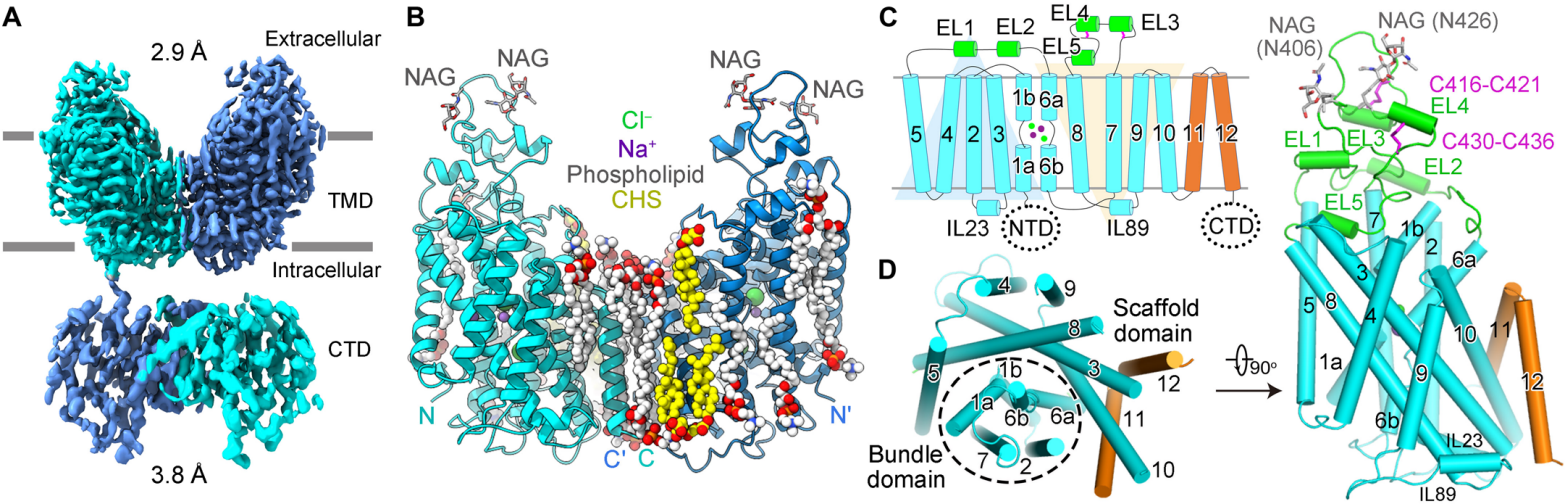
Cryo-EM structure of human NCC. (**A**) Overview of the cryo-EM structure of human NCC, with each subunit colored in cyan and marine. Transmembrane domain (TMD) and C-terminal domain (CTD) were determined at 2.9 and 3.8 Å, respectively. (**B**) Cartoon representation of TMD dimer in the same view as the cryo-EM map in (A). *N*-acetylglucosamine (NAG) is shown in gray sticks. Phospholipids and cholesteryl hemisuccinate (CHS) are shown as white and yellow spheres, respectively. Two Na^+^ and two Cl^−^ are shown as spheres in each subunit, colored in purple and green, respectively. (**C**) Topology arrangement of NCC, with ECD, TM1-10, and TM11-12 colored in green, cyan, and orange, respectively. The background of inverted repeats of TM1-5 and TM6-10 are shown in marine and yellow, respectively. (**D**) Structure of one TMD subunit in intracellular (left) and side (right) views, colored the same as in (C). Two NAG and two pairs of disulfide bonds are labeled as sticks, colored in gray and magenta, respectively. The numbers of transmembrane helices are indicated, with TM1, TM2, TM6, and TM7 forming the bundle domain, and TM3, TM5, TM8, and TM10 forming the scaffold domain. EL, extracellular loop; IL, intracellular loop.

We cloned human NCC gene using human embryonic kidney (HEK) 293 complementary DNA (cDNA) as template, obtaining an isoform of NCC. Compared with the NCC isoform UniProt ID: P55017-1, the NCC that we used in this study carries a naturally occurring mutation of A264G with Gln^95^ missing. For the convenience of mutual comparison analysis, we adopt the residue number of P55017-1 in the description of our structure. Full-length human NCC, with C-terminal His and Flag tags, was transiently expressed in HEK293F cells. After succeeding purification through tandem affinity and size exclusion chromatography (SEC), NCC was eluted in a single monodisperse peak and collected for cryo-EM single-particle analysis (fig. S1, A and B). For details of protein purification, data acquisition, and structural determination, please see Materials and Methods. Eventually, a cryo-EM map at an overall resolution of 2.9 Å for the transmembrane domain (TMD) of NCC was obtained out of 382,235 particles by C2 symmetry (Fig. 1A and figs. S1 and S2). The C-terminal domain (CTD) of NCC shows substantial conformational flexibility (fig. S1, C and D) and was determined at a moderate resolution of 3.8 Å (Fig. 1A and fig. S3) from 79,255 particles by C1 symmetry (table S1).

While the moderate resolution of the CTD did not support accurate reliable model building for side chains, the atomic model of CTD was docked as a rigid body based on the predicted model generated by SWISS-MODEL using Protein Data Bank (PDB) ID: 6NPL as template. The high resolution of NCC-TMD EM map enabled accurate atomic model building for the TMD and extracellular domain (ECD). Of note, the nonprotein densities corresponding to two Na^+^ and two Cl^−^ ions are seen. Hence, structural and functional analysis for the TMD of NCC is the focus of our study, which will be discussed below.

### Transmembrane domain

The overall NCC reveals dimeric assembly and displays domain-swap organization. Each subunit contains 12 transmembrane helices (TMs) with both the N- and C-terminal ends on the intracellular side (Fig. 1A and fig. S3). The TMD subunit adopts a LeuT-fold conformation, characterized by inverted repeats within TM1 to TM5 and TM6 to TM10 via a pseudo-twofold symmetry axis parallel to the membrane (Fig. 1C). Of note, TM1 and TM6 are broken at the lipid bilayer’s middle region, forming discontinuous sites suitable for ion binding (Fig. 1, C and D). Like other wild-type (WT) CCCs, our NCC structure is also trapped in an inward-open conformation occluded with substrate ions (Fig. 1, B and D). With the extracellular gate closed, the ion translocation pathway in our NCC structure extends from the center of the transmembrane region to the cytoplasmic side.

The ECD lying atop TMD is composed of two ordered linkers between TM5-6 and TM7-8, wherein two helices (EL1 and EL2) between TM5 and TM6 and three helices (EL3 to EL5) between TM7 to TM8 are formed (Fig. 1, C and D). Two glycosylation sites (Asn^406^ and Asn^426^) were observed with covalently linked N-acetylglucosamine moieties of sugar (Fig. 1D), essential for efficient expression and activity of NCC (*29*). Notably, EL3 and EL4 are stabilized by two pairs of disulfide bonds Cys^416^-Cys^421^ and Cys^430^-Cys^436^, respectively (Fig. 1D). Previous studies have shown that mutation of Cys^421^ to Arg in NCC caused Gitelman syndrome (*30*) and mutations of corresponding cysteines in KCC2 reduced its transport activity (*31*), suggesting the critical functions of these disulfide bonds in CCCs. Transport activity measurement for C421R NCC mutant by cell-based fluorescent quench method indicates nearly abolished transport activity (fig. S4) (see Materials and Methods). Compared to WT, the surface expression level and thermal stability of C421R mutant are hindered (fig. S5), suggesting the important function of Cys^421^ in efficient expression, maturation, and activity of NCC.

**Fig. 2.**
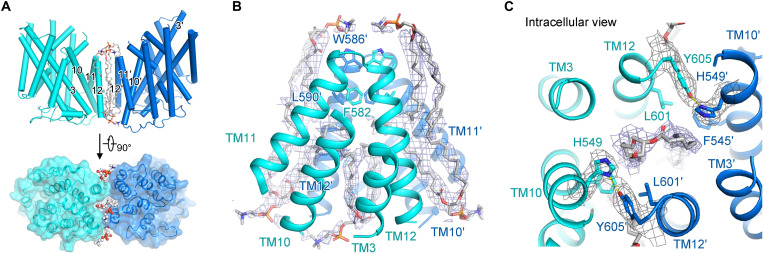
Lipids and TMD dimer interface of NCC. (**A**) Cartoon and surface representation of TMD in the side view (top) and the intracellular view (bottom), decorated with modeled lipids in the TMD dimer interface, with two TMD subunits colored in cyan and marine, respectively. (**B** and **C**) The ordered lipid molecules at the dimer interface are represented in the side view (B) and the intracellular view (C). Lipids are shown as white sticks, and lipid densities are shown as meshes (5σ). One lipid inserts into the intracellular cavity of the TMD dimer interface. In addition, four lipids bind to the side clefts of TMD dimer interface.

Multiple lipid densities were observed in our structure. A putative phospholipid inserts into the hydrophobic cavity formed by TM11 and TM12, together with four additional putative phospholipids, contributing to dimer interactions (Fig. 2, A and B). The polar interaction formed by His^549^ from TM10 and Tyr^605^ from the opposing subunit may also stabilize the dimerization (Fig. 2C). Compared with other amino acid–polyamine–organocation (APC) transporters such as LeuT and vSGLT (*32*, *33*), NCC retains relatively loose packing in the TMD dimer interface, as described in other CCCs (*18*, *19*). Besides, on account of the high resolution of TMD, more lipid densities in the shape of cholesteryl hemisuccinate (CHS) or phospholipids were shown (fig. S6), potentially participating in TM stabilization (*21*).

### Relative motions of CTD and TMD

CTD connects to TMD through a scissor helix (fig. S3, B and D). The dimerization contacts of NCC mainly derive from the extensive interactions of the two CTD subunits, as described in other CCCs (*18*, *21*–*23*, *25*–*28*). The twofold axes of the TMD and CTD dimers present slight distinction, with rotation of ~11° and tilt of ~27° between them (fig. S3C). Furthermore, structural comparisons of inward-open CCCs reveal a similar overall arrangement but with various motions of CTD relative to TMD (fig. S7). With TMD well aligned, from the intracellular view, CTDs of NCC and NKCC1 rotate ~11° and 30° in the counterclockwise direction, respectively. However, CTD of Na^+^-independent KCCs displays clockwise rotation (fig. S7). In addition, compared to KCCs and NKCC1, the CTD of NCC displays translational motions (fig. S7, B and D). Distinct motion directions of CTDs lead to similar arrangements between TM12 and scissor helix in NCC and NKCC1 but distinct in KCCs (fig. S7, E and F), implying diverse regulation mechanisms used in CCCs (*26*).

### The substrate ion binding sites

**Fig. 3.**
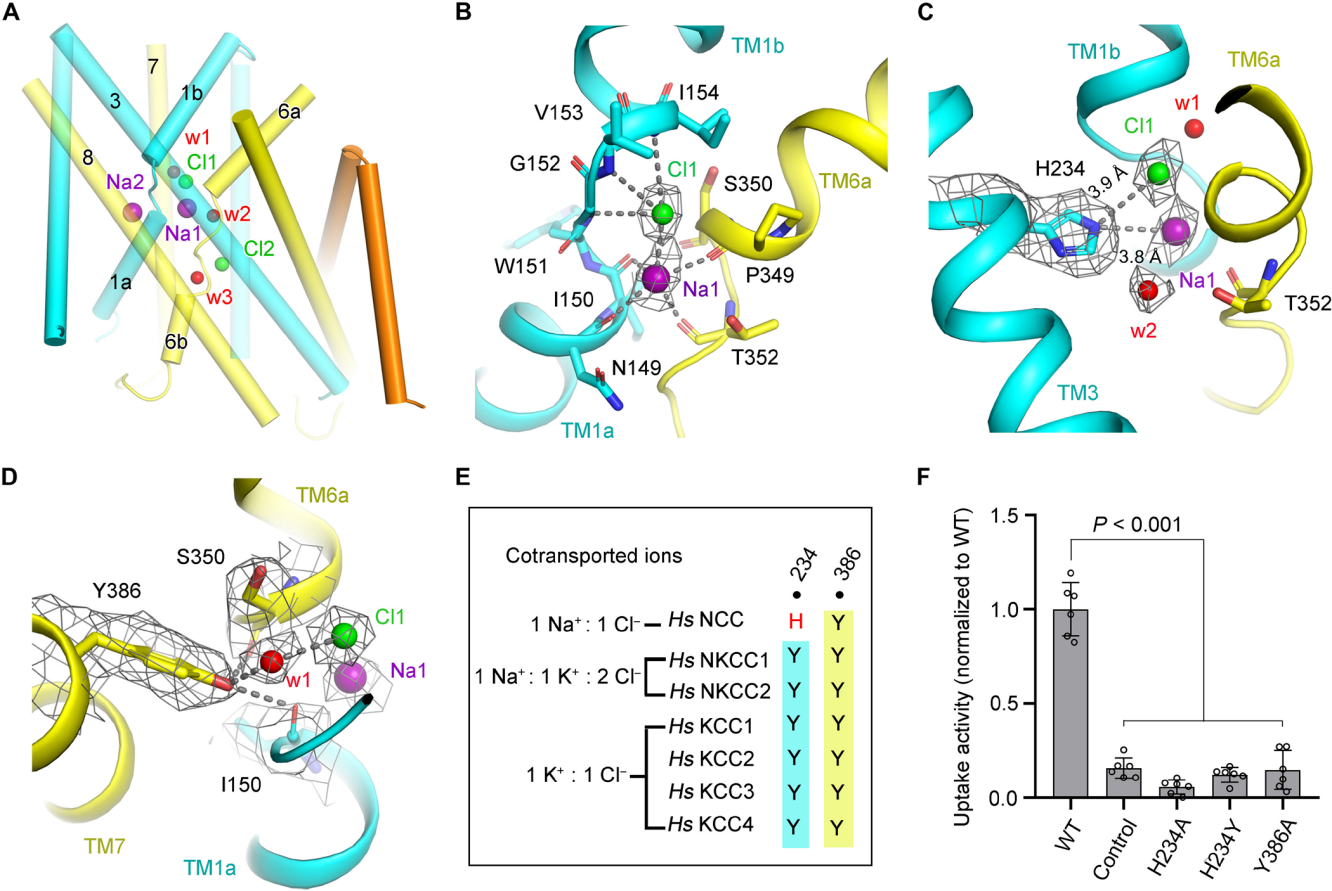
The Na1 and Cl1 at substrate binding sites of NCC. (**A**) The overall view showing the binding sites of Na1, Na2, Cl1, and Cl2 and three proposed water molecules in one TMD subunit. Na^+^, Cl^−^, and water molecules are individually indicated as purple, green, and red spheres. TM1-5, TM6-10, and TM11-12 are colored in cyan, yellow, and orange, respectively. (**B** and **C**) The close-up views of the Na1 and Cl1 at the substrate binding sites in the NCC. Cl1 and Na1 bind at the broken helices of TM1 and TM6, coordinated by the main-chain amide or carbonyl groups, respectively (B). His^234^ potentially coordinates Cl1 and Na1 and is proximal to one proposed water molecule (w2) (C). The densities corresponding to ions, water, or key residues are shown as mesh (5σ). (**D**) Tyr^386^ interacts with main-chain carbonyl groups of Ile^150^ and Ser^350^ and mediates coordination of Cl1 through a proposed water (w1). (**E**) Sequence alignment of His^234^ and Tyr^386^ in CCCs. NCC contains a His coordinating Na^+^, wherein Tyr is conserved to coordinate K^+^ in other CCCs. (**F**) Uptake activity determination of NCC with mutations (H234A, H234Y, and Y386A). The activity is normalized to the WT transporter (WT) (means ± SEM, *n* = 6 independent experiments). Control indicates empty vector control.

On the basis of the structures of NKCC1 and KCCs, the high resolution of NCC-TMD leads us to unambiguously accommodate two Na^+^ and two Cl^−^ in our structure (Fig. 3A). In addition, three extra nonprotein densities are also observed in the ion transport pathway, proposed to be water molecules considering the surrounding chemical environment (Fig. 3A). Because NCC transports Na^+^ and Cl^−^ with a ratio of 1:1, it is stoichiometrically reasonable to assign waters based on the existence of two Na^+^ and two Cl^−^. Notably, the third water site is exposed to the cytosolic solvent, providing a chance for a water molecule to reside in this site. Consistently, cotransport of water by CCCs has been reported in previous studies of NKCC1 and KCCs (*34*–*36*).

One Na^+^ and one Cl^−^, assigned as Na1 and Cl1, are located in the substrate pocket formed by the antiparallel broken helices of TM1 and TM6, corresponding to the conserved binding sites of K^+^ and Cl1 in NKCC1 and KCCs (*18*, *19*, *21*, *23*). Na1 and Cl1 coordinate each other with Cl1 sitting atop Na1. Cl1 is mainly coordinated by the main-chain amide group of Gly^152^, Val^153^, and Ile^154^ from the discontinuous break of TM1. The hydroxyl group of Ser^350^ also potentially supplies weak coordination for Cl1 (Fig. 3B). Besides, one water molecule, named w1, participates in interaction with Cl1, wherein a similar water site has also been recently described in human NKCC1 (*21*). Sandwiched by both the negative ends of dipole helices of TM1a and TM6a, Na1 is well accommodated by main-chain carbonyl groups from Asn^149^, Ile^150^, Pro^349^, and Thr^352^ (Fig. 3B). Of note, mutations of NCC (I150M, V153M, I154F, and P349L) can cause Gitelman syndrome, suggesting the vital role of these residues in the transport activity of NCC (table S2) (*2*, *12*, *37*).

Notably, the unique His^234^ from TM3, which is replaced by a Tyr in other CCCs, is at a distance of 3.8 and 3.9 Å from Na1 and Cl1, individually, indicating potential coordination for the substrate ions (Fig. 3, C and E, and fig. S8). Thus, considering that NCC is the unique member of CCC family that does not transport K^+^, this particular His^234^ residue might play a key role in proper Na^+^ substrate selection. In the substrate pocket, one proposed water molecule (designated as w2) was found to be proximal to Na1 and His^234^, potentially functioning in coupling for ion transport. Another conserved residue, Tyr^386^ from TM7, interacts with main-chain carbonyl groups of Ile^150^ and Ser^350^ from broken helices of TM1 and TM6, respectively. Tyr^386^ mediates the coordination of Cl1 through the water molecule w1 (Fig. 3D). Because Tyr^386^ is highly conserved in all CCCs (Fig. 3E), our structure indicates that Tyr^386^ plays an essential role in ion coordination and potential conformational coupling involving TM1, TM6, and TM7 during transport. Notably, H234Q and Y386C led to Gitelman syndrome, further indicating the prominent roles of these two residues in NCC activity (table S2) (*30*).

Cell-based fluorescent quench transport activity assays were performed to detect the function of these residues in NCC. The mutation of Tyr^386^ to Ala abolished transport (Fig. 3F and figs. S4 and S5), indicating the important function of Tyr^386^ in transport of NCC. Moreover, His^234^ was mutated to Ala, Tyr, and a Gitelman-related mutation, Gln, individually, showing abolished transport activity for H234A and H234Y and moderately reduced transport activity for H234Q compared to WT NCC (Fig. 3F and fig. S4). However, the protein stability of all three His^234^ mutants are affected, in terms of expression yield, maturation [altered glycosylation pattern on the SDS–polyacrylamide gel electrophoresis (PAGE)], and stability (SEC profiles, clear aggregation for H234Y) compared to the WT NCC, suggesting the critical role of His^234^ in protein stability and transport activity of NCC linked to posttranslation modification (fig. S5).

### The Cl2 and Na2 binding sites

**Fig. 4.**
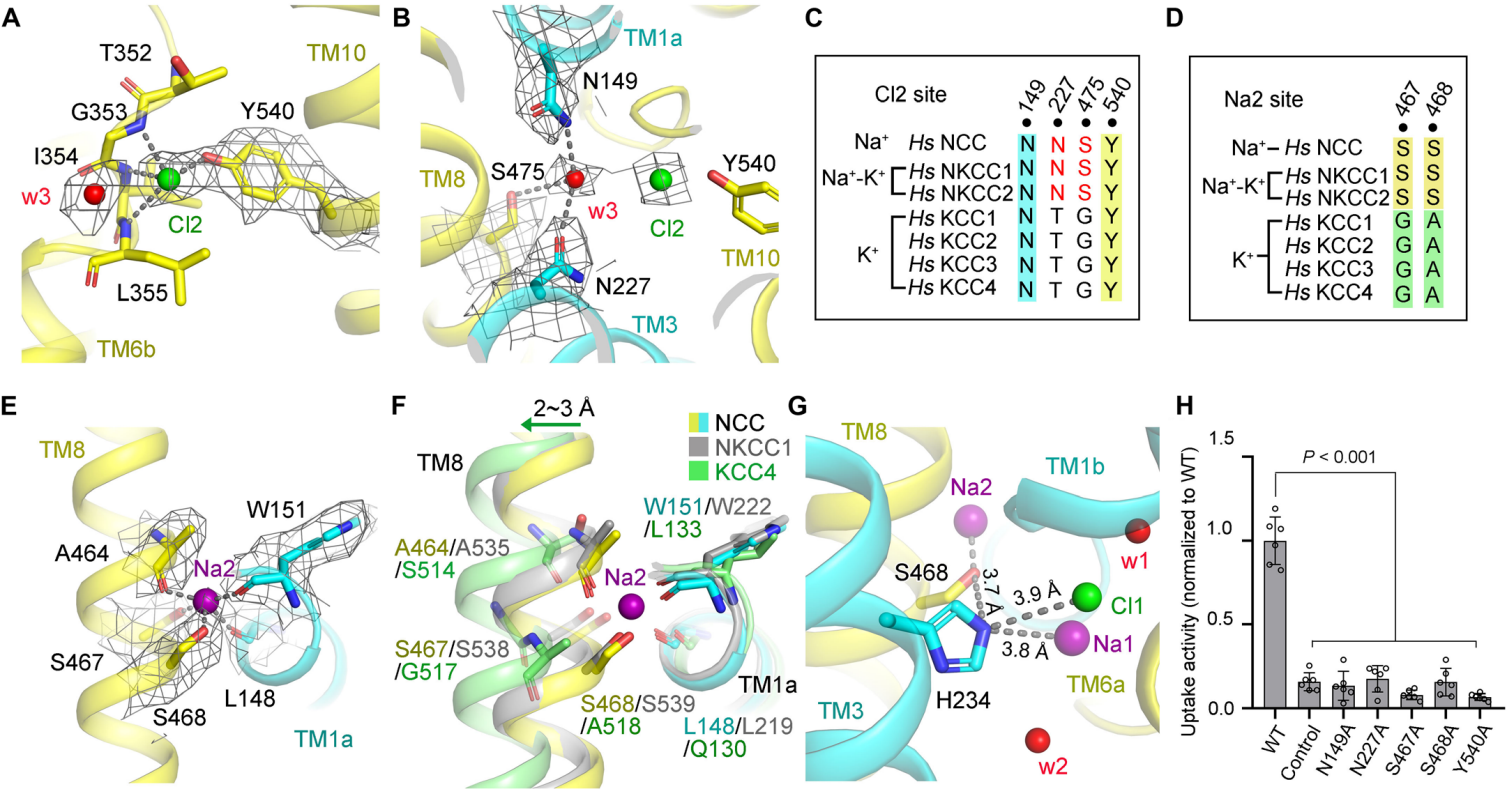
The ion binding sites of Cl2 and Na2 in NCC. (**A**) The Cl2 ion binding sites in NCC. Cl2 is coordinated by three main-chain amide groups from the broken helix of TM6 and the hydroxyl group of Tyr^540^, proximal to a water molecule (w3). Na^+^, Cl^−^, and water molecule are indicated as spheres, colored in purple, green, and red, respectively. Residues involved in ion coordination are shown as sticks. The densities corresponding to ions, water, or key residues are shown as mesh (5σ). (**B**) Nearby Cl1, a water molecule (w3) interacts with Asn^149^, Asn^227^, and Ser^475^. (**C**) Sequence alignment of the Cl2 site of CCCs. (**D**) Sequence alignment of the Na2 binding site of CCCs. Two Ser residues are conserved in Na^+^-dependent CCCs (NCC, NKCC1, and NKCC2) but absent in K^+^-dependent KCCs. (**E**) The Na2 ion binding sites in NCC. (**F**) Structural superimpositions of human NCC (this study), zebrafish NKCC1 (PDB ID: 6NPL, gray), and human KCC4 (PDB ID: 7D99, green) at the Na2 binding site in the same orientation as shown in (A). The TM8 of KCC4 shifts away for 2 to 3 Å compared to NCC or NKCC1. (**G**) Distance display of His^234^ to surroundings, indicating that His^234^ might potentially mediate the coordination of Na2 through Ser^468^. (**H**) Uptake activities of NCC with mutations (N149A, N227A, S467A, S468A, and Y540A). The activity was normalized to the WT transporter (WT) (means ± SEM, *n* = 6 independent experiments, WT and control are the same as in Fig. 3F). Control indicates empty vector control.

In CCCs, the Cl2 binding site is highly conserved (*38*–*40*), which was also found in our structure. In NCC, Cl2 is consistently coordinated by main-chain amide groups (Gly^353^, Ile^354^, and Leu^355^) from the broken helix of TM6 and the hydroxyl group of Tyr^540^, a conserved residue from TM10 (Fig. 4, A and C). A putative water density (designated as w3) exposed to the cytosolic solvent is proximal to Cl2 (Fig. 4, A and B). Water w3 interacts with side chains of Asn^149^, Asn^227^, and Ser^475^ from TM1a, TM3, and TM8, respectively (Fig. 4B). Note that Asn^149^ is conserved in CCCs, whereas Asn^227^ and Ser^475^ coordinating this putative water are particularly conserved in Na^+^-dependent CCCs, which are replaced by Thr and Gly, respectively, in KCCs (Fig. 4C). Mutations of N149A or N227A significantly reduced the transport activity of NCC (Fig. 4H and figs. S4 and S5). Besides, a previous study demonstrated that S475C only retained 40% residual Na^+^-uptake activity of NCC (*41*). Moreover, the mutant of Tyr^540^ to Ala nearly abolished transport activity of NCC, implying the important roles of Tyr^540^ in transport (Fig. 4H and figs. S4 and S5).

In addition to Na1, another Na^+^ binding site (designated as Na2) was observed in NCC, as described in NKCC1 (*18*, *21*, *27*). This Na2 site is conserved in Na^+^-dependent LeuT-fold APC transporters, such as LeuT, SiaT, and MhsT (fig. S9) (*32*, *42*, *43*). In NCC, Na2 is coordinated by hydroxyl groups of Ser^467^ and Ser^468^ from TM8, and main-chain carbonyl groups of Ala^464^, Trp^151^, and Leu^148^ (Fig. 4E). Further sequence alignment shows that two Ser residues in Na2 site are conserved in Na^+^-dependent CCCs (NCC, NKCC1, and NKCC2) but absent in Na^+^-independent KCCs (Fig. 4D), implying the particular roles of these two serine residues in Na^+^ selection and coordination at Na2 site. In comparison to structures of NCC and NKCC1, TM8 of KCCs shifts for 2 to 3 Å away from the Na2 site, leading to the absence of Na2 site in KCCs (Fig. 4F) (*19*, *23*). Notably, His^234^, the particular residue, which might coordinate Na1 and Cl1 as mentioned above, was observed to potentially mediate Na2 coordination through Ser^468^, suggesting that His^234^ plays a vital function in the coordination of ions in NCC (Fig. 4G). Furthermore, mutations of the two conserved Ser residues to Ala individually led to the clear reduction of ion transport of NCC, demonstrating the important function of these two Ser during transport (Fig. 4H and figs. S4 and S5).

### An inward-open conformation of NCC

**Fig. 5.**
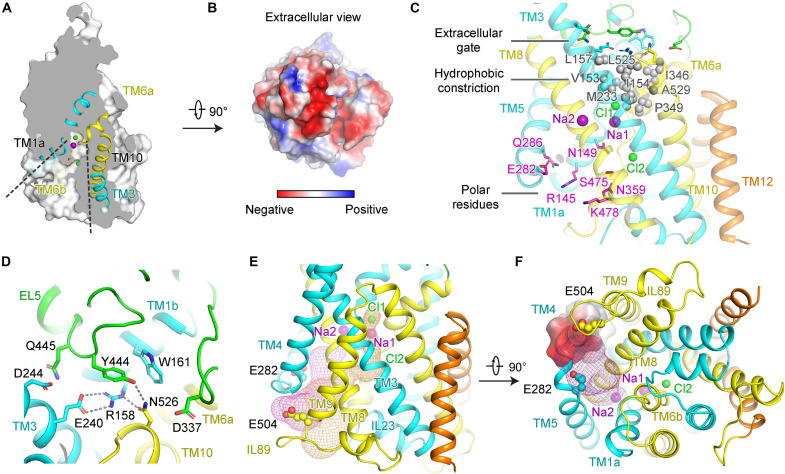
An inward-open conformation of NCC. (**A**) Slab view of one TMD showing the cytoplasmic access cavity, indicating an inward-open conformation with ion substrate occluded. (**B**) The extracellular side of NCC presents electronegativity. (**C**) The extracellular gate, hydrophobic constriction atop ion binding sites, and a series of inward-open polar residues in NCC. Extracellular gate and polar residues are shown as sticks, with the inward-facing polar residues colored in magenta. Residues involved in hydrophobic constriction are shown as gray spheres. (**D**) The extracellular gate of NCC mediated by a salt bridge and polar interactions. (**E**) The proposed transport pathways for substrate ions (Na1 and Cl1) and Na2, generated by HOLE, are shown in orange and magenta meshes, respectively. (**F**) Proposed Na2 transport pathway lining TM5. Na2 is probably attracted by intracellular electronegative regions formed by the electronegative end of TM4 dipole and nearby residues of Glu^282^ and Glu^504^ from TM5 and IL89, respectively.

Our structure is trapped in an inward-open conformation occluded with ion substrates (Fig. 5). The extracellular gate is closed mainly through a conserved salt bridge in CCCs (*38*), which is formed between Arg^158^ and Glu^240^ in NCC. Polar interactions derived from Tyr^444^ and Asn^526^ further strengthen the close of the extracellular gate (Fig. 5, C and D). A loss-of-function E289G variant of mouse KCC3 has indicated that this salt bridge plays a vital role in transport (*44*). Nonpolar residues, sandwiched by the extracellular gate and substrate ion pocket, form a hydrophobic constriction, further effectively hindering the escape of ions back into the extracellular side (Fig. 5C).

The inward-open cavity in the TMD of NCC is encircled by amounts of polar residues, composing the transport exit pathway for Na^+^ and Cl^−^ into the cytoplasm (Fig. 5C). Intracellular-facing transport pathways generated by HOLE (*45*) reveal two major exit pathways for Na1, Cl1, and Cl2, mainly lining TM6b and TM5, respectively (Fig. 5E, orange). Notably, underlying the Na2 site, residues of Glu^282^ and Glu^504^ from TM5 and intracellular loop 89 (IL89), respectively, and the C-terminal electronegative end of TM4 dipole form an electronegative region, which might attract and escort Na2 to exit into the cytoplasm lining TM5. Thus, we generated a proposed pathway for Na2 (Fig. 5, E and F, magenta), as indicated in NKCC1 (*20*, *21*).

### Structural comparisons between inward-open NCC and outward-open NKCC1

**Fig. 6.**
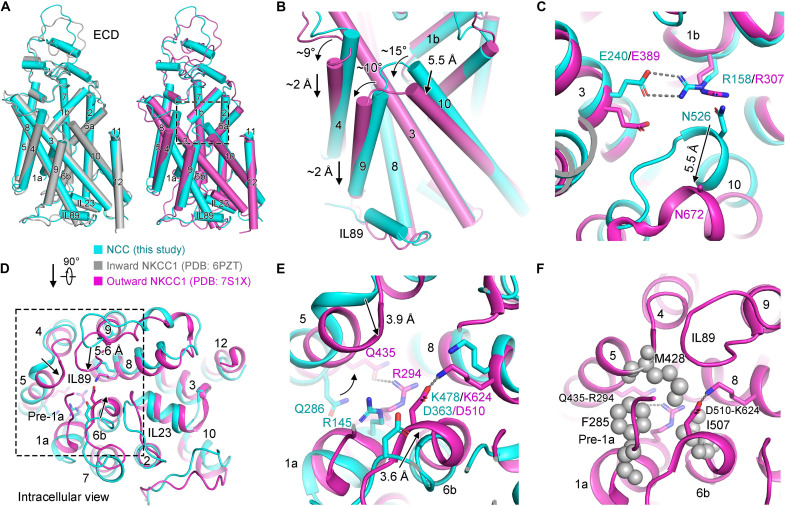
Structural comparison of inward-open NCC and outward-open NKCC1. (**A**) Structural superimpositions of inward-open human NCC (this study) and inward-open human NKCC1 (left, PDB ID: 6PZT), and outward-open human NKCC1 (right, PDB ID: 7S1X, bound with bumetanide), colored in cyan, gray, and magenta, respectively. (**B**) Conformational changes of TM4, TM9, and TM10 between inward-open NCC and outward-open NKCC1. (**C**) Conformational transitions of TM3 and TM10 from inward-open to outward-open state lead to the break of the salt bridge and polar interactions in the extracellular gate, shown in a magnified view boxed in right in (A). (**D**) Intracellular view of structural comparison between inward-open NCC and outward-open NKCC1 reveals significant interior movements of IL89, TM6b, and connection between TM4 and TM5, shifting for 5.6, 3.6, and 3.9 Å, respectively, along with the newly ordered pre-TM1a loop, constricting the inward-facing cavity in outward-open NKCC1. (**E**) In the cytoplasmic cavity, two pairs of salt bridges are formed when transferring from inward-open to outward-open state, shown in a zoomed-in view boxed in (D). (**F**) In addition to the two pairs of salt bridges as displayed in (E), the intracellular gate is further closed through steric hindrance derived from IL89 and pre-TM1a loop and a concurrent hydrophobic constriction formed by Met^428^, Phe^285^, and Ile^507^.

Recently, an outward-open human NKCC1 structure bound with bumetanide has been reported (*27*). Structural comparisons of inward-open human NCC with inward or outward-open NKCC1 both reveal similar overall architectures, with a root mean square deviation of 0.58 Å over 294 Cα atoms within one TMD subunit between NCC and the inward-open human NKCC1 (PDB ID: 6PZT) (*18*) and 1.44 Å over 304 Cα atoms between NCC and the outward-open human NKCC1 (PDB ID: 7S1X) (*27*) (Fig. 6A). On the basis of the high similarity of NCC and the inward-open NKCC1, we analyzed structural comparisons of our inward-open NCC with the reported outward-open NKCC1. As a result, their overall architectures reveal minor distinctions, wherein multiple slight conformational changes emerge (Fig. 6A, right).

The N termini of TM10 from outward-open NKCC1 shift for 5.5 Å and tilt for ~15°, resulting in the dilation of the extracellular gate. Concomitantly, the movement of TM10 might trigger a ~10° slight tilt and ~2 Å downward shift to the cytoplasm of TM9, followed by a similar movement of TM4, causing further dilation of the extracellular gate (Fig. 6B). Consequently, as supposed previously (*27*), because of fine-tuning of TM3 and the significant shift of TM10, the salt bridge and polar interaction in the extracellular gate become broken in the outward-open state (Fig. 6C).

In contradiction to the dilated extracellular gate, the cytosolic-facing cavity in TMD of outward-open state is constricted by interior ingression of IL89, TM6b, and TM4-TM5 linker, shifting for 5.6, 3.6, and 3.9 Å, respectively, compared to inward-open NCC, accompanied by the newly ordered pre-TM1a loop (Fig. 6D). Because of the slight downward shift and rotation of TM9, the interior ingression of IL89 blocks the cytoplasmic-facing transport pathway in the outward-open state. Benefitting from conformational transitions of TM5 and TM6b, two pairs of salt bridges at Gln^435^-Arg^294^ and Asp^510^-Lys^624^ are generated in outward-open NKCC1, whereas the corresponding residues are distant in NCC (Fig. 6E). In addition, steric obstruction of the newly observed pre-TM1a loop and concurrent nonpolar constriction formed by hydrophobic interactions derived from Met^428^, Phe^285^, and Ile^507^ further enclose the intracellular gate (Fig. 6F).

## DISCUSSION

Here, we report the structure of human NCC in the inward-open state, illustrating the ion binding sites (fig. S10A). Compared to reported NKCC1 and KCCs structures, our structure presents the conserved substrate ion sites (Na1 and Cl1) and the Cl2 binding site (fig. S11) (*38*–*40*). As to the Na^+^-dependent transporter, Na2 is observed in our structure, which has also been proposed with weak intensity in NKCC1 (*18*, *27*). Notably, as a unique member that does not transport K^+^ in CCCs, a particular His residue in substrate pocket is observed to interaction with Na1 and Cl1 and might also participate in the coordination of Na2 through a Ser, indicating the potential function of His in Na^+^ selection and transport. However, the substitution of His^234^ in NCC to the corresponding conserved Tyr in other CCCs does not enable NCC to transport K^+^ (*38*), suggesting that the mechanism of ion selection of CCCs is complicated, which requires further studies.

Speculated from structural superimposition, the transformation from inward- to outward-open state of CCCs mainly involves interior ingressions of intracellular loops (IL89 and pre-TM1a) and the formation of two pairs of salt bridges and a hydrophobic constriction to close the intracellular gate, and concurrently, the dilated movement of N termini of TM10 and the break of a conserved salt bridge might facilitate the opening of the extracellular gate. Distinct from the overall significant conformational changes of TM1a-1b and TM6a-6b indicated in LeuT (*32*, *46*), the relatively slighter movements of CCC TMs likely enable much faster transport turnover of CCC, as proposed in NKCC1 and KCC1 (*27*, *28*).

Multiple lipids are observed in our structure, among which one lipid binds to the N termini of TM10, one lipid interacts with TM9, and a putative cholesterol lies near the N termini of TM6a, wherein these three TMs reveal major movements between inward- and outward-open CCCs. Similar cryo-EM densities have been observed in inward-open NKCC1 (*21*) but were absent in the outward-open NKCC1 (*27*). As indicated in a recent study of NKCC1 (*21*), we infer that lipids might be involved in stabilizing the TMs of CCCs in the inward-open conformation. To investigate the transport mechanism of NCC more precisely, the outward-open structure of NCC remains to be solved.

Because of the critical roles of NCC in maintaining the homeostasis of sodium, many disease mutations of NCC associated with Gitelman syndrome have been reported (*41*, *47*–*51*). We mapped these disease mutations into the NCC structure by categorizing them into several groups based on their locations or functions in NCC (fig. S10B and table S2). Thus, our structure establishes a framework for interpreting the extensive disease-related mutations in Gitelman syndrome. Considering NCC as the vital drug target for hypertension, our structure promotes the further development of cardiovascular drugs.

## MATERIALS AND METHODS

All resources and primer sequences used in this study are shown in table S3 and S4, respectively.

### Protein expression and purification

Human NCC (UniProt ID: P55017-1) cDNA was cloned into a pCAG vector containing a C-terminal tandem affinity tags (His tag and FLAG tag), using HEK293 cDNA as template and obtaining a construct with naturally occurring A264G mutation with Q95 missing due to the coexistence of several isoforms of NCC in cells. For convenience of mutual comparison, we adopt the residue number of P55017-1 in this study. NCC protein was expressed in HEK293F cells (Life Technologies). To express NCC, cells were cultured in SMM 293-TII medium (Sino Biological Inc.) at 37°C under 5% CO_2_ at 130 rpm in a Multitron-Pro shaker (Infors), transiently transfected with the plasmid and polyethylenimines (PEIs) (Polysciences) when the cell density reached approximately 2.0 × 10^6^ cells/ml. For transfection of 1 liter of cell culture, 2 mg of plasmids was premixed with 4 mg of PEIs in 40 ml of fresh medium for 15 min before adding them into cell culture. The transfected cells were cultured for 48 hours.

For purification of WT and mutant NCC, cells were harvested by centrifugation at 2000*g* and resuspended in the buffer containing 25 mM tris (pH 8.0), 150 mM NaCl, aprotinin (1.3 μg/ml; Amresco), pepstatin (0.7 μg/ml; Amresco), and leupeptin (5 μg/ml; Amresco). The membrane fraction was extracted with 2% (w/v) *n*-dodecyl-β-d-maltopyranoside (Anatrace) at 4°C for 2 hours. After extraction, the supernatant was collected by centrifugation at 12,000*g* for 1 hour (JA-25.50, Beckman Coulter); loaded to anti-FLAG M2 affinity resin (Sigma-Aldrich); washed in buffer containing 25 mM tris (pH 8.0), 150 mM NaCl, 0.01% (w/v) lauryl maltose neopentyl glycol (Anatrace), and 0.01% (w/v) CHS (Anatrace) and eluted with buffer containing 25 mM tris (pH 8.0), 150 mM NaCl, 0.02% (w/v) glyco-diosgenin (GDN; Anatrace), and FLAG peptide (0.4 mg/ml). The protein sample was concentrated and further purified by SEC on a Superose 6 10/300 GL column (GE Healthcare) using Bio-Rad NGC QuestTM 10 Plus in buffer containing 25 mM tris (pH 8.0), 150 mM NaCl, and 0.02% (w/v) GDN. The protein peak fraction was determined by SDS-PAGE gel and collected for cryo-EM analysis.

### Cryo-EM sample preparation and data acquisition

For cryo-sample preparation, the purified NCC protein was concentrated to 9 mg/ml. Four microliters of NCC protein was applied to a glow-discharged Quantifoil R1.2/1.3 300-mesh gold holey carbon grid, blotted for 3.5 s, and flash-frozen in liquid ethane cooled by liquid nitrogen with Vitrobot (Mark IV, Thermo Fisher Scientific).

Micrographs were acquired on a Titan Krios microscope (FEI) operated at 300 kV with a K2 Summit direct electron detector (Gatan) and GIF Quantum energy filter. SerialEM (*52*) software was used for automated data collection following the standard procedures. A nominal magnification of ×81,000 was used for imaging, yielding a pixel size of 1.046 Å on images. The defocus range was set from −1.5 to −2.3 μm. Each micrograph was dose-fractionated to 36 frames with a total exposure time of 3.009 s, resulting in a total dose of about 52.8 *e*^−^/Å^2^. A total 5897 movie stacks were collected.

### Cryo-EM data processing

The data processing pipeline is shown in (fig. S1C). The motion correction was performed using patch motion correction (multi), and the CTF parameters of the micrographs were estimated using patch CTF estimation (multi) in cryoSPARC v3.2 (*53*). Then, 4254 good micrographs were manually selected from 5897 movies. Particles were blob and template-picked and subjected to two-dimensional (2D) classification to generate initial models. A total of 2,180,112 particles were automatically picked and extracted with a binning factor of 2 and subjected to 3D classifications with the class number (*K*) equaled to 3, 4, or 5.

For generating the final map of NCC TMD region, 3D classes including 1,970,041 particles showing good secondary structural features in TM region (boxed in red, fig. S1C, left) were selected and combined, reextracted for bin 1 particles, followed by several rounds of hetero-refinement, CTF refine, and nonuniform refinement with C2 symmetry. The final 3D reconstruction of NCC TMD region from 382,235 particles yielded an EM map with a resolution of 2.85 Å (table S1). 2D classification determination shows apparent features of TMD but fuzzy features of CTD in this dataset (fig. S1D).

For generating the overall map of NCC, 3D classes including 1,236,162 particles showing good structural features of both TMD and CTD (boxed in blue, fig. S1C, right) were selected and combined, reextracted for binning 1 particles, followed by several rounds of 3D classifications both in cryoSPARC (*53*) and Relion 3.1 (*54*), CTF refine, and nonuniform refinement with C1 symmetry. The final 3D reconstruction of the overall NCC from 79,255 particles yielded an EM map with a resolution of 3.75 Å (table S1). To increase the resolution of each part of the overall map, local mask was applied during the last nonuniform refinement on the TMD or CTD, yielding EM maps with a resolution of 3.66 and 3.77 Å for TMD and CTD (fig. S1C), respectively. 2D classification determination shows distinguished CTD features after the above data processing for overall map (fig. S1E).

### Model building

The atomic model of NCC was built using the predicted model generated by SWISS-MODEL (*55*) (template: PDB ID: 6NPL) as a starting template. Atomic model building based on the 2.85 Å resolution density map of NCC TMD was performed in Coot (*56*), refined in real space using Phenix.real_space_refine (*57*) with Ramachandran and NCS restraints. Validation tools in Phenix (*57*) and MolProbity (*58*) were used to guide iterative rounds of model adjustment in Coot and refinement in Phenix. The 2.85 Å resolution density map allowed us to construct an NCC TMD model containing residues 137 to 605. The atomic model of CTD containing residues 616 to 1020 was docked as a rigid body based on the predicted model generated by SWISS-MODEL and refined by Phenix (table S1). All figures were prepared in PyMOL (www.pymol.org) or Chimera (*59*).

### NCC transport activity measurements using eYFP-mKate

Uptake activity of NCC WT and mutants was measured by I^−^ influx using HEK293T cotransfected with mKate and enhanced yellow fluorescent protein (eYFP)–NCC constructs. The halide transport activity of NCC quenches the fluorescence of eYFP while having no effect on mKate. Thus, the transport activity is linked to the variation of the eYFP/mKate ratio. Intracellular halide was measured as previously reported (*60*, *61*) with some modifications. HEK293T cells were cotransfected with the halide-sensitive pCAG-Flag-eYFP-NCC constructs or pCAG-Flag-eYFP vector as control and pCAG-mKate construct using PEIs in a 1:2 (w/w) ratio. The total amount of DNA per transfection was 2 μg (0.2 μg of mKate and 1.8 μg of eYFP-NCC constructs) and added in a 20-mm-diameter plate with a density of 1.5 × 10^6^ cells/ml. After 36 hours, cells were transferred to a hypotonic low-chloride buffer [70 mM Na gluconate, 4.2 mM KCl, 1.4 mM CaCl_2_, 1 mM MgCl_2_, 5.5 mM d-glucose, and 10 mM Hepes (pH 7.4)] for 10 min at 37°C. NCC-expressing HEK293T cells were identified by detecting eYFP fluorescence using a monochromator at 488-nm excitation. After 30 s, an equal volume of NaI solution [140 mM NaI, 4.2 mM KCl, 1.4 mM CaCl_2_, 1 mM MgCl_2_, 5.5 mM d-glucose, and 10 mM Hepes (pH 7.4)] was added to each dish. The final concentration of Na^+^ and I^−^ was 105 and 70 mM, respectively. Changes in intracellular halide concentrations were monitored by simultaneous measurements of halide-sensitive eYFP fluorescence and the fluorescence of mKate. eYFP was excited at 488 nm by using a monochromator, and the emitted light measurement ranged from 493 to 530 nm. mKate was excited at 543 nm, and the emitted light measurement ranged from 592 to 650 nm. Changes in fluorescence intensity were monitored by a Leica SP8 confocal microscope, and images were recorded every 15 s. After measurement, quantitative image analysis was performed with ImageJ. All statistical analyses were performed using one-way analysis of variance (ANOVA) with Tukey’s multiple comparisons test comparing NCC-WT with mutants.

### Western blotting

HEK293T cells transiently transfected with NCC WT and mutants were used for activity measurement and then washed three times with phosphate-buffered saline before collection. The collected cells were then lysed by mixing with SDS loading buffer and loaded onto a 16% (w/v) SDS-PAGE gel. After electrophoresis, transfer, and blot, membranes were probed with anti–Flag–horseradish peroxidase antibody (GNI) and anti–β-actin (CWBIO). The membrane was visualized with the imaging system (ChemiDoc Touch; Bio-Rad, Hercules, CA, USA) using enhanced chemiluminescence (ECL) (Smart-Lifesciences).

### Thermal shift assay

The Prometheus NT.48 nanoDSF (NanoTemper Technologies) was used for the analysis of thermostability in our research. Each sample was filled into the capillaries and with at least three replicates. The capillaries were heated from 20° to 95°C with a heating rate of 1°C/min. Fluorescence ratio (F350/F330) was monitored to determine the apparent melting temperature (*T*_m_) during a nanoDSF scan. The nanoDSF data analysis was performed using PR.ThermControl v2.1.5 software (NanoTemper Technologies). The statistical analysis was executed using the GraphPad Prism 8.0.

## Notes

View/request a protocol for this paper from *Bio-protocol*.
