# Supplementary material for: Cryo-EM structure of the human sodium-chloride cotransporter NCC
Source: Sci Adv. 2022 Nov 9;8(45):eadd7176. doi: 10.1126/sciadv.add7176 (PMC9645730; doi:10.1126/sciadv.add7176)
Supplement: Supplementary file 1 — Figs. S1 to S11 Tables S1 to S4 [file sciadv.add7176_sm.pdf]

Science Advances
AAAS

Supplementary Materials for

Cryo-EM structure of the human sodium-chloride cotransporter NCC

Jing Nan *et al.*

Corresponding author: Yanqing Zhang, zyq_lab@fudan.edu.cn

Sci. Adv. **8**, eadd7176 (2022)
DOI: 10.1126/sciadv.add7176

This PDF file includes:

Figs. S1 to S11
Tables S1 to S4

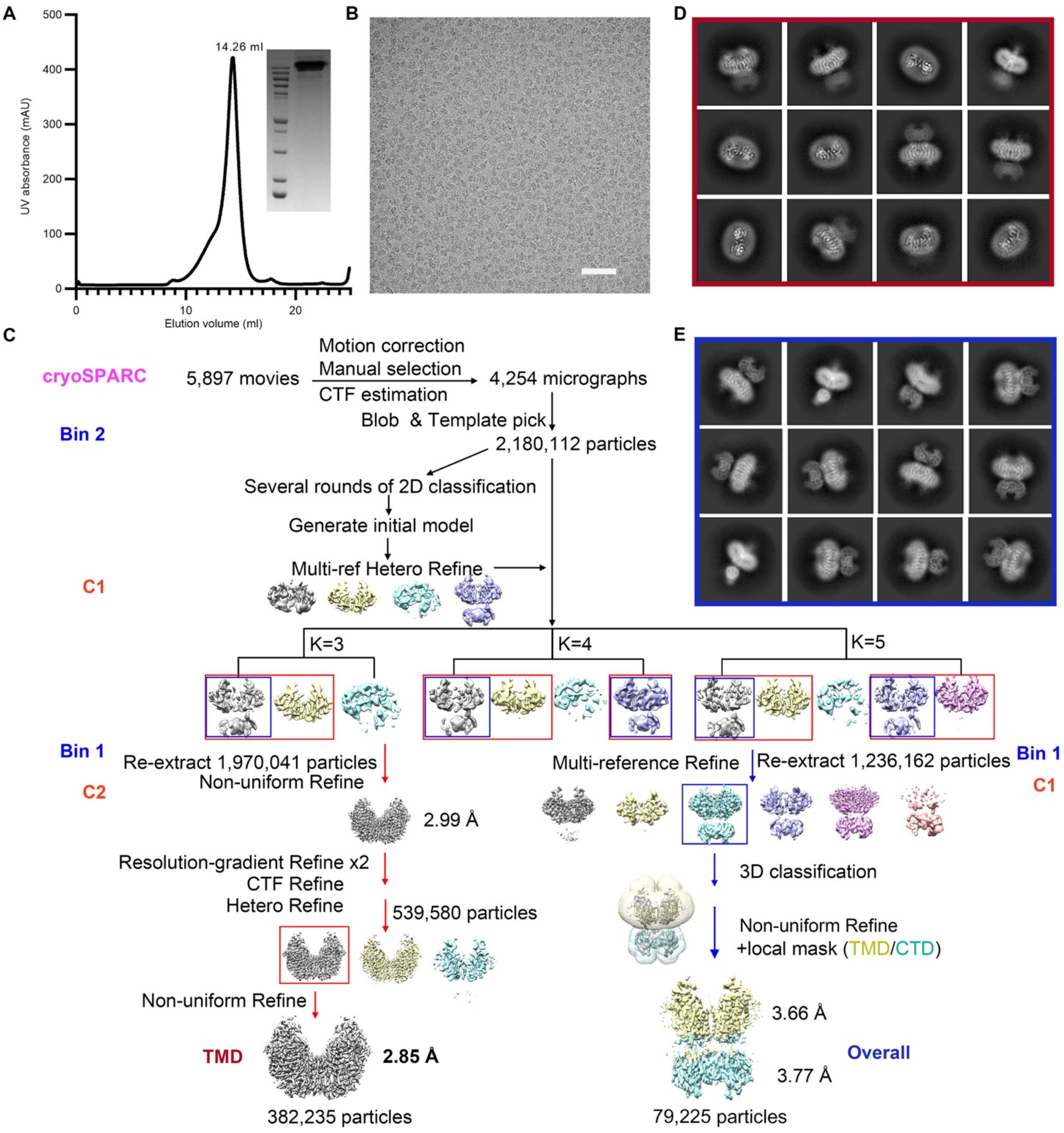

Fig. S1. Biochemical characterization and cryo-EM data processing workflow for NCC. (**A**) Size-exclusion chromatography of NCC by Superose 6 (GE Healthcare) and the protein peak detected by SDS-PAGE gel. (**B**) A representative cryo-EM micrograph. Scale bar: 50 nm. (**C**) Flowchart of image processing for final cryo-EM maps of TMD (left, boxes or arrows in red) and CTD (right, boxes or arrows in blue). (**D**) Representative 2D classes generated from particles for NCC-TMD map. High-resolution features are shown in the TMD region, whereas the CTD is fuzzy, indicating conformational flexibility in CTD. (**E**) Representative 2D classes generated from particles for overall NCC map. Features of the CTD are distinguished after extensive data processing for the CTD.

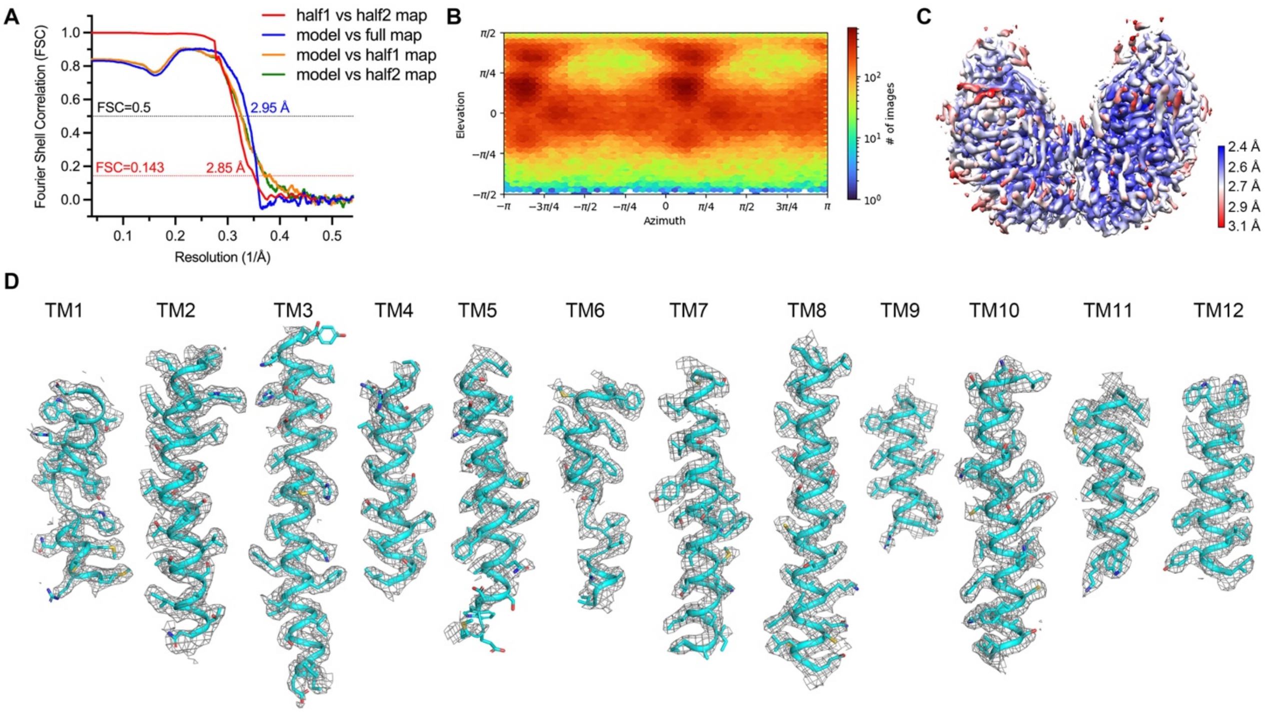

Fig. S2. 3D reconstruction analysis, statistics, and representative density maps of the TMD of NCC. (**A**) Fourier Shell Correlation (FSC) curves. Gold-standard FSC curve between two half maps with indicated resolution at 0.143 (red); FSC curve for cross validation between the atomic model and the final TMD map with indicated resolution at 0.5 (blue); FSC curve between the atomic model refined against half 1 map and half 1 map (orange) or half 2 map (green). (**B**) Angular distributions for particles contributing to the cryo-EM map of TMD. (**C**) The local resolution map of TMD. (**D**) The cryo-EM density maps for all transmembrane helices shown as mesh (5σ), with atomic models shown as cartoon and colored in cyan.

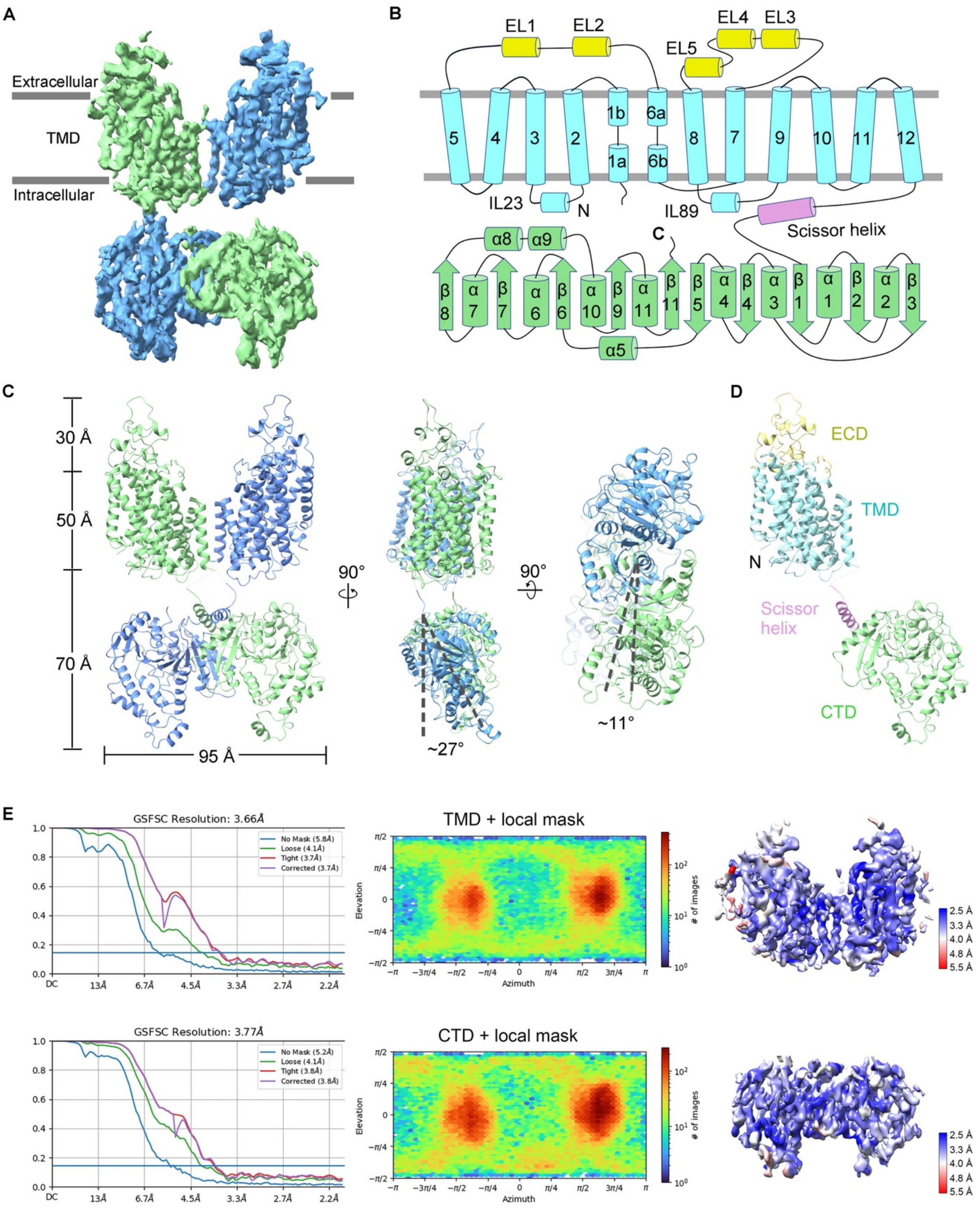

Fig. S3. Overall structure of NCC. (**A**) Side view of a three-dimensional reconstruction of NCC with each subunit colored in green and marine. (**B**) Topology and domain arrangement of the NCC subunit. ECD, TMD, and CTD are colored in yellow, cyan, and green, respectively. (**C**) Cartoon representation of the NCC dimer in the same orientation as in (A). (**D**) The structure of NCC subunit in side view. Each domain is colored the same as in (B). (**E**) FSC curve, particle angular distribution, and local resolution map of TMD (upper) and CTD (lower) during overall structure determination of NCC.

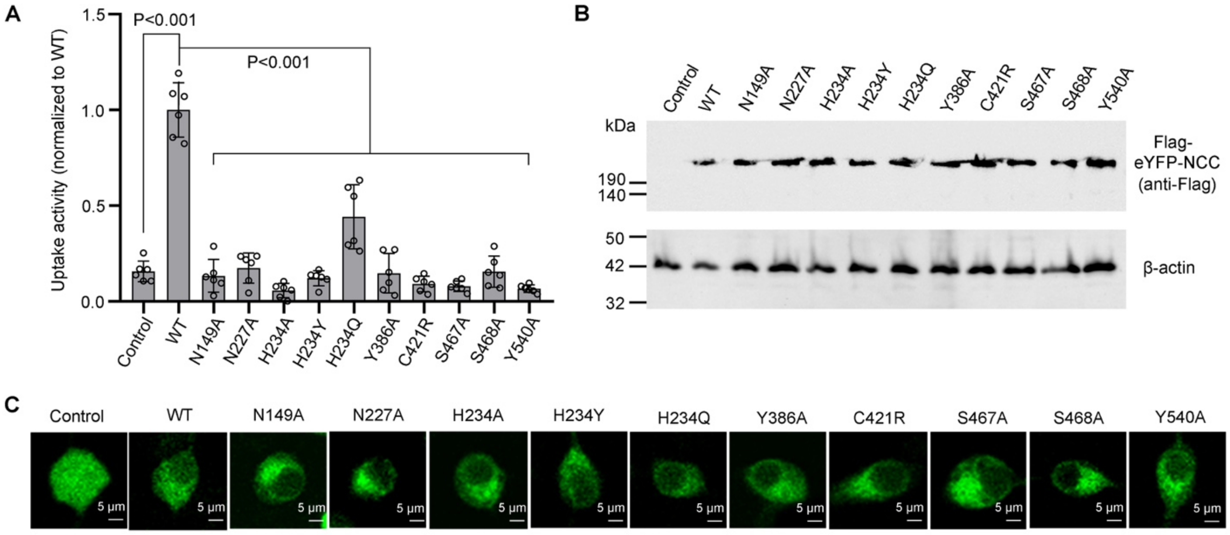

Fig. S4. Transport activity measurement for wild-type and mutant NCC by cell-based fluorescent quench method. (**A**) Uptake activities measurements of wild-type and mutant NCC. The activity was normalized to the wild-type transporter (WT) (mean ± s.e.m., n=6 independent experiments). Control indicates empty vector control. (**B**) Western blotting detection of total protein expression level of WT NCC and mutants from HEK293T cells. Immunoblots were probed with anti-Flag, with β-actin as sample loading control. (**C**) Representative fluorescent images of cells expressing eYFP-tagged NCC WT and mutants.

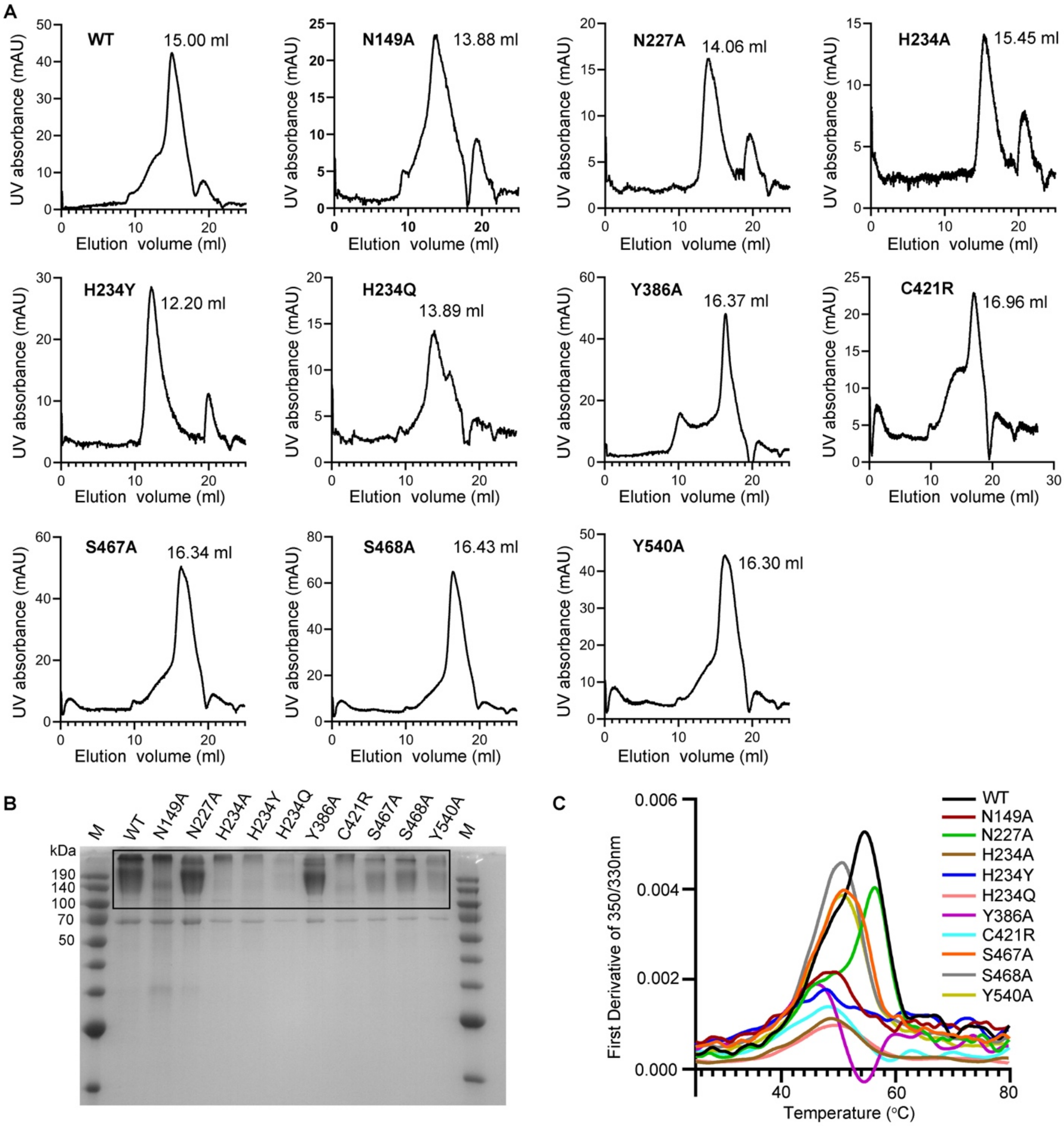

Fig. S5. Size-exclusion chromatography and thermal shift assay for wild-type and mutant NCC. (**A**) Size-exclusion chromatography of wild-type (WT) NCC and mutants. Peak volume is indicated. (**B**) SDS-PAGE analysis of WT NCC and mutants. Boxed are the bands of NCC (smear relates to glycosylation of NCC). (**C**) Differential scanning fluorimetry (DSF) analysis of WT NCC and mutants.

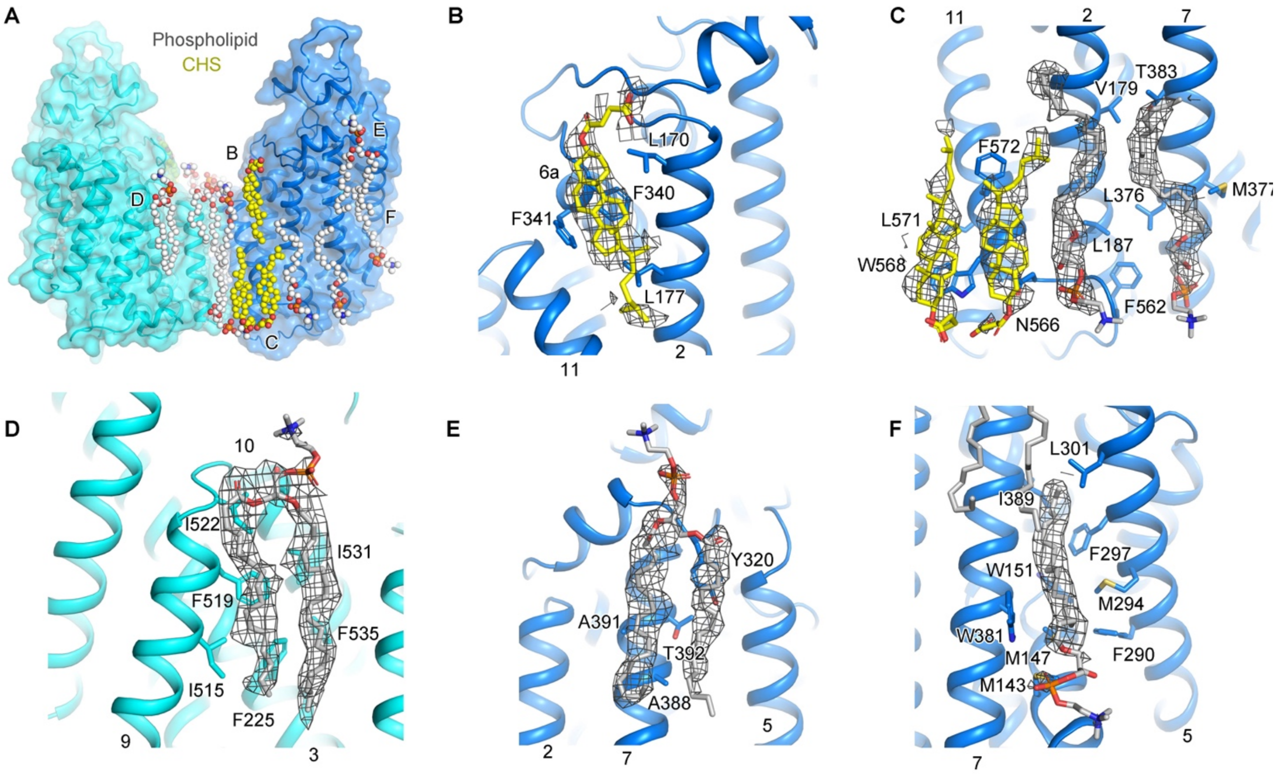

Fig. S6. Lipids at TMD of NCC. (**A**) Cartoon and surface representations of TMD in side view, decorated with proposed lipids shown as spheres, with phospholipids and CHS (cholesteryl hemisuccinate), colored in white and yellow, respectively. (**B-F**) The magnified views of CHS or phospholipid binding sites at the N-termini of TM6 (B), TM11 (C), TM10 (D), C-termini of TM7 (E), and the cavity between TM5 and TM7 (F), respectively. Lipids are shown as sticks, and densities are revealed as meshes (5σ).

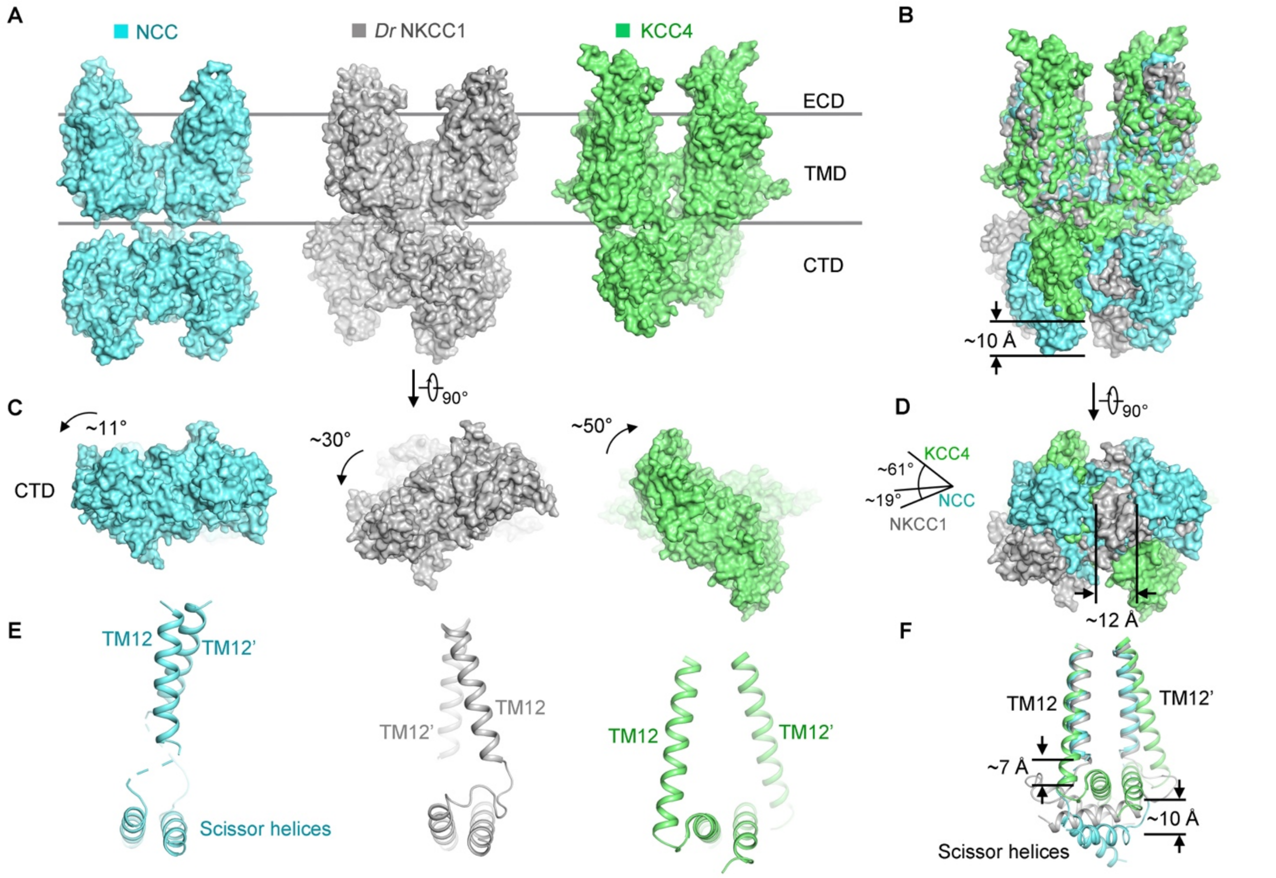

Fig. S7. Motions between the CTD and TMD of CCCs. (**A**) Side view of the surface maps of human NCC (this study, cyan), *Danio rerio* (*Dr*) NKCC1 (PDB ID: 6NPL, gray), and human KCC4 (PDB ID: 7D99, green). (**B**) Structural superimpositions of NCC (cyan), *Dr* NKCC1 (gray), and KCC4 (green) with the TMD dimer aligned. (**C**) Intracellular view of the surface maps of NCC, *Dr* NKCC1, and KCC4. (**D**) Intracellular view of structural superimpositions of NCC, *Dr* NKCC1, and KCC4 with the TMD dimer aligned. (**E**) Distinct structural arrangement of the TM12 and scissor helices in NCC (cyan), *Dr* NKCC1 (gray), and KCC4 (green), with the scissor helices aligned. (**F**) Structural comparisons of the TM12 and scissor helices in NCC, *Dr* NKCC1, and KCC4, with the TMD aligned in the similar view as in (B).

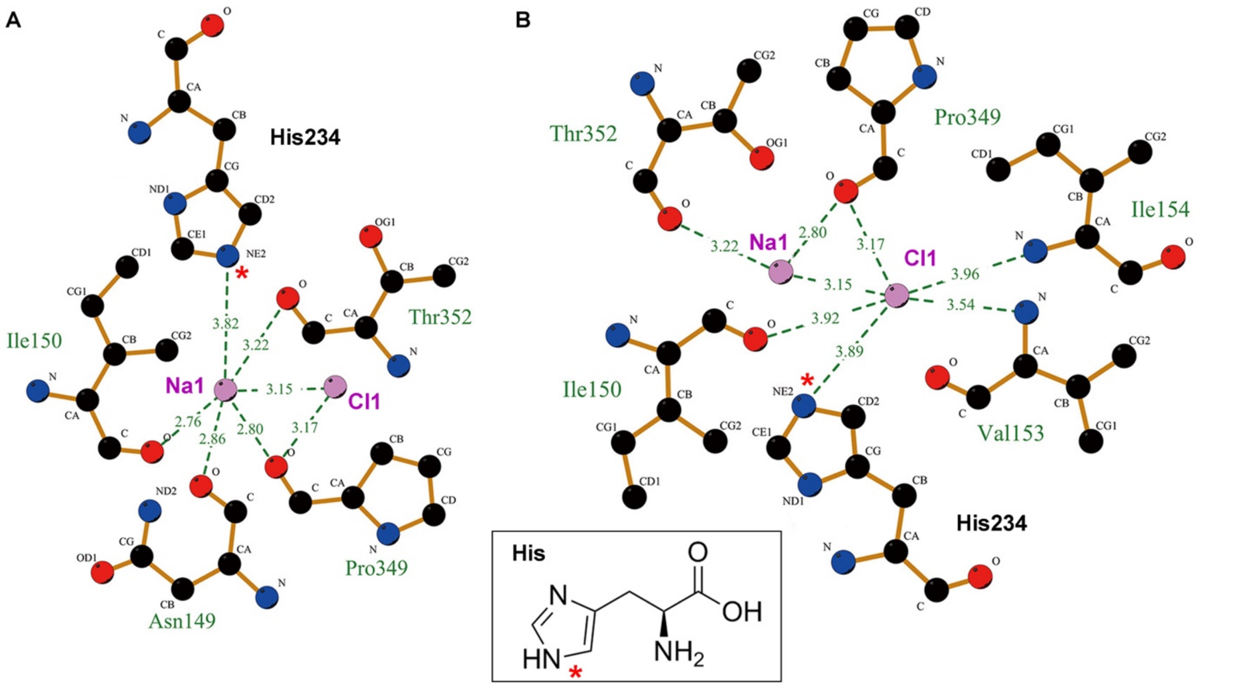

Fig. S8. LigPlot Plus analysis for the interactions of His234 with Na1 and Cl1 in NCC. (**A**) Potential interaction of His234 with Na1 ion is indicated by dashed lines between the atoms involved. (**B**) Potential interaction of His234 with Cl1 ion is indicated by dashed lines between the atoms involved. The structural formula of histidine is displayed in the bottom inset. The imino group in the 5-member ring of His234 is indicated by a red asterisk, which potentially participate in the coordination of Na1 or Cl1.

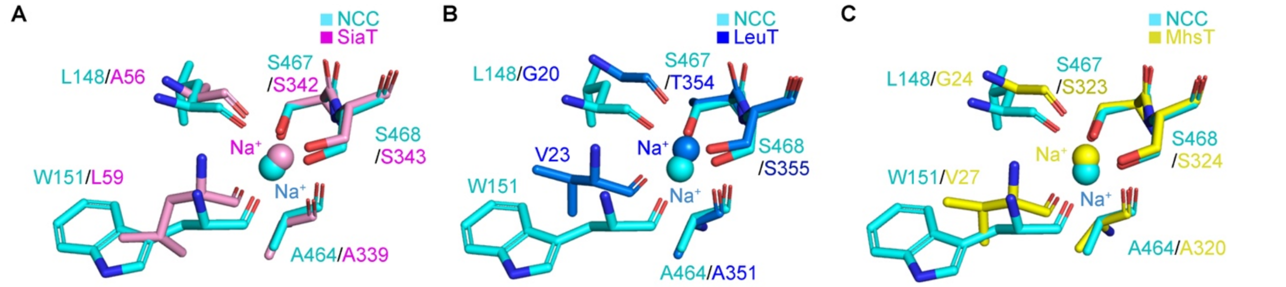

Fig. S9. Structural comparisons of the conserved Na2 binding site. Structural superimpositions of the conserved Na2 binding sites between human NCC (this study, cyan) and *Pm* SiaT (PDB ID: 5NVA, pink) (**A**), *Aa* LeuT (PDB ID: 2A65, marine) (**B**), or *Ah* MhsT (PDB ID: 4US3, yellow) (**C**), respectively. *Pm*, *Proteus mirabilis*; *Aa*, *Aquifex aeolicus*; *Ah*, *Alkalihalobacillus halodurans*.

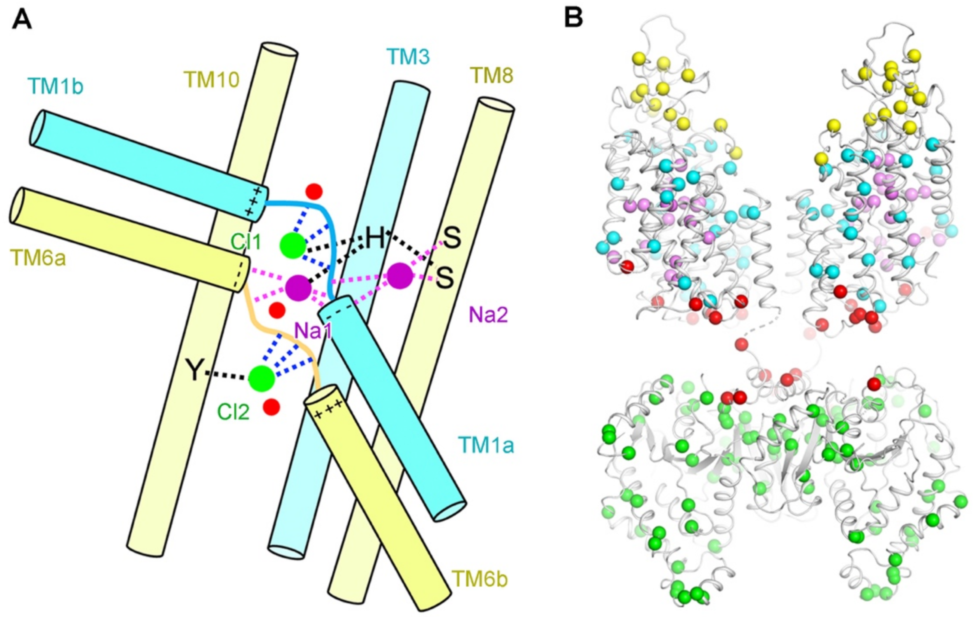

Fig. S10. Schematic ion binding sites and Gitelman syndrome-related mutations in NCC. (**A**) Schematic of ion binding sites and putative waters in NCC transporter. Helices are shown as cylinders with coordination interactions shown as dashed lines. Na^{+}, purple; Cl^{-}, green; water, red. (**B**) Gitelman syndrome-linked mutations are mapped onto the structure of NCC, related to Table S2. Mutations are indicated as spheres and categorized into five groups with different colors according to their locations. ECD, yellow; ion-translocation pathway, pink; other TMD mutations, cyan; TMD-CTD interface, red; and mutations in CTD, green.

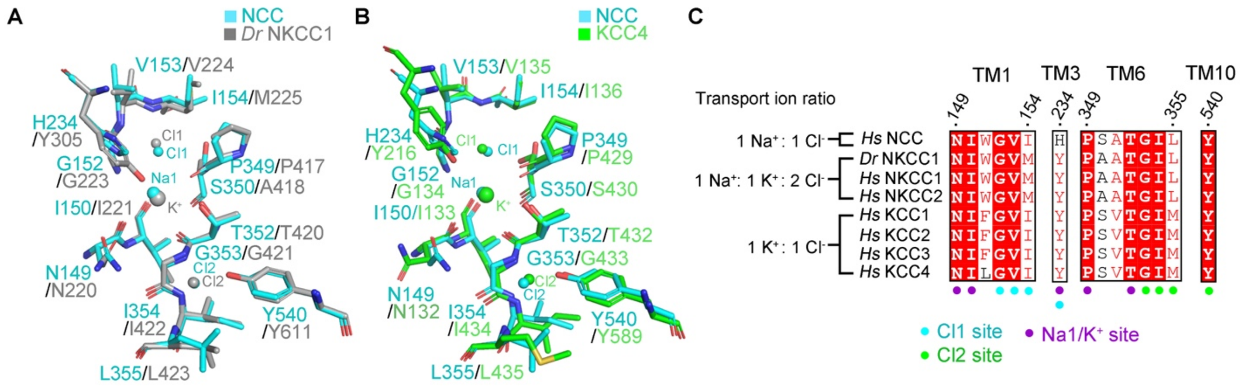

Fig. S11. The conserved ion binding sites in CCCs. (**A-B**) Structural superimpositions of the substrate (Na1/K^+ and Cl1) and Cl2 binding sites between human NCC (this study, cyan) and *Dr* NKCC1 (PDB ID: 6NPL, gray) (A) or human KCC4 (PDB ID: 7D99, green) (B). (**C**) Sequence alignment around the conserved ion binding sites among CCCs. The residues coordinating ions are indicated with circles below the sequence. Cyan, Cl1 binding sites; green, Cl2 binding sites; purple, Na1/K^+ binding sites.

Table S1. Cryo-EM data collection and refinement statistics.

	NCC-TMD	NCC-CTD	NCC-overall
**Data collection**			
EM equipment		Titan Krios	
Voltage (kV)		300	
Detector		Gatan K2	
Energy filter		20 eV	
Pixel size (Å)		1.046	
Electron dose ($e^-/Å^2$)		52.8	
Defocus range (μm)		-1.5 to -2.3	
Number of collected movie stacks		5,897	
**Reconstruction**			
Software		cryoSPARC	
Number of used particles	382,235	79,255	79,255
Symmetry	C2	C1	C1
Overall resolution (Å)	2.85	3.77	3.75
Map sharpening B- factor ($Å^2$)	-137.3	-127.7	-100.5
**Model building and refinement**			
Software		Phenix & Coot	
Model composition			
Protein residues	938	770	1708
Side chains assigned	934	768	1702
Na	4	0	0
Cl	4	0	0
POV	9	0	0
LPE	8	0	0
Y01	6	0	0
NAG	6	0	0
Water	6	0	0
R.m.s. deviations			
Bond length (Å)	0.014	0.005	0.005
Bond angles (°)	2.109	1.150	1.071
Ramachandran plot statistics (%)			
Favored	96.68	91.60	91.98
Allowed	3.21	8.40	8.02
Outlier	0.11	0.00	0.00
**EMDB**	33641	33804	33803
**PDB**	7Y6I	7YG1	7YG0

Table S2. Disease mutation statistics of NCC in Gitelman syndrome.

Group name	Mutation site
Ion-translocation pathway	R145C/H, I150M, V153M, I154F, L157P, R158L, A226T, G230D, H234Q, T235R, S283Y, P349L, N359K, Y386C, G463E, A464T, S475C, K478E
Extracellular domain	A313V, S314, G316V, R321W, R334W, T339C, R399C, S402F, N406H, C421R, N426K, C430G, G439S, N442S
Other TM mutations	T163M, A166V, W172R, S178L, T180K, G186D, I192T, T194I, L215P, D259N, R261H, G264A, L272P, K284R, A285G, T304M, G342A, G374V, T382M, T392I, A459D, C484W, R507C, A523T, N534S, F536L, L542P, S546G, A569E, A570V, L571P, V578M, A588V
TM-soluble interface mutations	G196V, R209Q, D486N, Y489H, G496C, S555L, P560H, S561Missing, N566Missing, S615W, L623P, G630V, S824T, R852C/H/S, R854
Other CTD mutations	Y641, R642C/G/H, V647M, T649R, R655H, M672I, V677I, A728T, G729V, G731R, P735R, L738R, G741R, P751L, D839N, L849F/H, L850P, R862C, G867S, R871H, M872T, R887Q, R904Q/N, R919C, R934W, R935W, R955Q, R958G, G980R, C985Y, R1009Q, Q1021R

Table S3. Key resources.

Materials and reagents	Source	Product catalogue
**Bacterial and virus strains**		
E. coli DH5α	Invitrogen	Cat#18265017
**Chemicals, peptides, and recombinant proteins**		
n-Dodecyl-β-D-Maltopyranoside (DDM)	Anatrace	Cat#D310
Cholesteryl Hemisuccinate Tris Salt (CHS)	Anatrace	Cat#CH210
Lauryl maltose neopentyl glycol (LMNG)	Anatrace	Cat#NG310
Glyco-diosgenin (GDN)	Anatrace	Cat#GDN101
SMM 293T-II medium	Sino Biological lnc.	Cat# SMM 293-T1
DMEM/High Glucose	Cytiva	Cat#SH30243.01
Anti-Flag M2 affinity gel	Sigma	Cat#A2220
Flag peptide	Sigma	Cat#F3290
Ampicillin	Amresco	Cat#69-52-3
Aprotinin	Amresco	Cat#E429
Pepstatin	Amresco	Cat#J583
Leupeptin	Amresco	Cat#J580
Trizma base	Sigma	Cat#77-86-1
40-kDa linear polyethylenimines (PEIs)	YEASEN	Cat#40816ES02
Superose 6, 10/300 GL	GE Healthcare	Cat#17-5172-0
50-kDa cut-off Centricon	Millipore	Cat#UFC910096
**Experimental models: Cell lines**		
HEK293F	GIBCO	Cat#R7900
HEK293T	ATCC	Cat#CRL-3216
**Recombinant DNA**		
Human NCC cloned onto a modified pCAG vector (His-Flag tag)	This study	N/A
Human NCC cloned onto a modified pCAG vector (N-terminal Flag and eYFP tag)	This study	N/A
mKate cloned onto a modified pCAG vector (C-terminal His-Flag tag)	This study	N/A
**Antibodies**		
Anti-DYKDDDDK (Flag)	GNI	Cat#GNI4310-FG
Anti-β-actin	CWBIO	Cat#CW0096
**Other**		
R1.2/1.3 300 mesh Au grids	Quantifoil	Q17080

Table S4. Primer sequences.

Primer	Sequence
12A3F	GAATTATCGATCCGGAGGTACCATGGCAGAACTGCCCACAAC
12A3R	CAACCTCATCAGAGCCCTCGAGCTGGCAGTAAAAGGTGAGCAC
FYFPF1	GAATTATCGATCCGGAGGTACCATGGTCGAGGATTACAAGGATGACGATG
FYFPF2	TACAAGGATGACGATGACAAGATGGTGAGCAAGGGCGAGGAGC
FYFPR	GGGCAGTTCTGCCATAGAGCCCTTGTACAGCTCGTCCATGCCG
R12A3F	GACGAGCTGTACAAGGGCTCTATGGCAGAACTGCCCACAACAG
R12A3R	CAACCTCATCAGAGCCCTCGAGTCACTGGCAGTAAAAGGTGAGCACG
mKateF	GAATTATCGATCCGGAGGTACCATGGTGAGCGAGCTGATTAAG
mKateR	CAACCTCATCAGAGCCCTCGAGCTATCTGTGCCCCAGTTTGCTAG
149F	GTGATGATTCGTTGCATGCTCGCAATTTGGGGCGTGATCCTCTAC
149R	GTAGAGGATCACGCCCCAAATTGCGAGCATGCAACGAATCATCAC
227F	GCCTCATTTTCGCTTTCGCCGCAGCCGTGGGTGTGGCCATGCAC
227R	GTGCATGGCCACACCCACGGCTGCGGCGAAAGCGAAAATGAGGC
234AF	CAATGCCGTGGGTGTGGCCATGGCAACGGTGGGCTTTGCAGAGACC
234AR	GGTCTCTGCAAAGCCCACCGTTGCCATGGCCACACCCACGGCATTG
234YF	CAATGCCGTGGGTGTGGCCATGTACACGGTGGGCTTTGCAGAGACC
234YR	GGTCTCTGCAAAGCCCACCGTGTACATGGCCACACCCACGGCATTG
234QF	GTGGGTGTGGCCATGCAGACGGTGGGCTTTGCAGAGACCGTG
234QR	TGCAAAGCCCACCGTCTGCATGGCCACACCCACGGCATTGGC
386F	GACCATTTCCGCACTGGCCATCTCAGCCACCATTG
386R	AGATGGCCAGTGCGGAAATGGTCGTCCAGAAAATG
421F	TGCGAGGGGCTGGCCCGAAGCTATGGCTGGAACTTCACCGAG
421R	GTTCCAGCCATAGCTTCGGGCCAGCCCCTCGCAGGCACCCCAG
467F	CTTCGGGGCCACCCTCGCCTCTGCCCTGGCCTGCCTTGTCTC
467R	GGCAGGCCAGGGCAGAGGCGAGGGTGGCCCCGAAGATGCCAG
468F	GGGGCCACCCTCTCCGCAGCCCTGGCCTGCCTTGTCTCTGCTG
468R	AAGGCAGGCCAGGGCTGCGGAGAGGGTGGCCCCGAAGATGCCAG
540F	CCTCTGCTCCGCAGCCCTCATCAACTTCAGCTGC
540R	TGATGAGGGCTGCGGAGCAGAGGAAGAAGTTGG
